# Trophoblast stem cells and syncytiotrophoblasts lack inflammatory responses to LPS but retain robust interferon-mediated antiviral immunity

**DOI:** 10.1530/RAF-25-0176

**Published:** 2026-07-07

**Authors:** Cristine R Camp, Joshua Baskaran, Matthew Brown, Carly Parker, Paige Drotos, Rachel C West

**Affiliations:** Anatomy, Physiology, Pharmacology Department, College of Veterinary Medicine, Auburn University, Auburn, Alabama, USA

**Keywords:** trophoblast stem cell, syncytiotrophoblast, LPS, interferon beta, innate immune response, sex differences

## Abstract

**Abstract:**

Early pregnancy requires a tightly regulated pro-inflammatory environment shared between the primitive placenta and decidua. While immune balance supports successful implantation and placental invasion, disruptions in immune signaling during this period can impair implantation and lead to embryo loss. In this study, we investigated the molecular mechanisms underlying immune imbalance during implantation using a trophoblast stem cell (TSC) model. TSCs were cultured in either stem cell or syncytiotrophoblast (STB) differentiation medium and treated with either lipopolysaccharides (LPSs) or interferon beta (IFNB). RT-qPCR and western blotting revealed that LPS failed to induce a pro-inflammatory cytokine response in TSCs or STBs. In contrast, IFNB triggered a strong antiviral response in both TSCs and STBs. RNA sequencing of IFNB-treated TSC and STB 3D spheroids revealed subtle differences between the TSC and STB responses to interferons. Both TSC and STB IFNB-treated spheroids mount an interferon-mediated antiviral response; however, STB spheroid genes associated with the type I interferon response, viral RNA/DNA sensing, and antigen processing were upregulated. We also compared the interferon response between the CT27 (female) and CT29 (male) TSCs and STBs. While STBs showed minimal differences, the CT29 TSCs exhibited a markedly stronger interferon response than the CT27 TSCs. Collectively, these findings suggest that the primitive placenta is selectively responsive to interferon signaling rather than direct pathogen-associated stimuli. This implies that maternal immune activation, rather than microbial invasion, likely drives that placental immune response and embryo success at this stage. Understanding these dynamics underscores the importance of the maternal immune balance in early pregnancy success.

**Lay summary:**

The maternal immune system has a direct influence on the success of embryo implantation. If that balance is disturbed, the placenta can fail to invade into the uterus and implantation can fail, leading to embryo mortality and pregnancy loss. In our study, we used stem cells from the placenta to explore how they respond to different kinds of immune signals (i.e. signals from bacteria versus signals from the mother’s immune response). We found that placental stem cells do not respond to signals from bacteria. However, they do react strongly to signals that resemble a viral infection in the mother. These data suggest that the early placenta is more sensitive to the mother’s immune signals than to direct infection, which highlights the critical role of maternal immune health in early pregnancy.

## Introduction

Early pregnancy requires the maintenance of a delicate balance of maternal immune signaling. At the onset of implantation, the fertilized egg has already reached the blastocyst stage and consists of the inner cell mass and the trophectoderm, the two cell lineages that will eventually give rise to the fetus and placenta, respectively. To initiate implantation, the trophectoderm adheres to and then breaches the epithelial layer of the decidualized endometrium (Ochoa-Bernal & Fazleabas 2020). Immediately following attachment to the decidualized endometrium, the trophectoderm fuses to form a highly invasive primitive syncytium that allows the embryo to breach the surface epithelium and invade into the underlying endometrium (Turco & Moffett 2019). Both the pre-implantation embryo and invasive primitive placenta help stimulate an immunological cascade coming from the immune cells that comprise the decidua (Mor *et al.* 2017). High levels of pro-inflammatory cytokines are necessary to support adequate trophoblast invasion and angiogenesis during early pregnancy (Sehring *et al.* 2022). However, dysregulated inflammatory responses driven by infection or autoimmunity can lead to impaired implantation and embryo loss (Mor *et al.* 2017). The peri-implantation period is a notoriously difficult time to study due to the scientific and ethical limitations surrounding human embryos and early pregnancy. Therefore, there is a gap in our understanding as to how the cells of the primitive placenta interact with the uterine immune milieu in both normal and pathological circumstances.

Human trophoblast stem cells (TSCs) (Okae *et al.* 2018) have recently become a widely used model to study early placental development. TSCs derived from both the trophectoderm of blastocysts and the villous column cytotrophoblast cells have similar transcriptome and DNA methylome signatures in all lines (Okae *et al.* 2018), suggesting the possibility of their use to study the formation of the primitive placenta. Furthermore, the transcriptomes of TSCs compared with the extended embryo culture, peri-implantation placental cells demonstrate similarities between TSCs and gestational day 8 cytotrophoblast cells (Logsdon *et al.* 2024), suggesting that TSCs model the early primitive cytotrophoblast. In addition, syncytiotrophoblasts (STBs) differentiated from TSCs are transcriptionally similar to the early STB described in gestational day 10 and 12 primitive placental cells (Logsdon *et al.* 2024). Another study documented that differentiating TSCs toward the syncytiotrophoblast (STB) led to the formation of an invasive STB capable of breaching the epithelium (Ruane *et al.* 2022), which is a hallmark of the primitive syncytium. Collectively, these data support the idea that TSCs are a novel model system capable of recapitulating early implantation dynamics.

Previous research using murine TSCs demonstrated that TSCs have attenuated immune responses to the inflammatory cytokines TNF and IFNG (Fendereski *et al.* 2024), suggesting that the primitive placenta can limit the cytotoxicity associated with a pro-inflammatory uterine environment. Fendereski *et al.* proposed that the early embryo is an ‘immune-privileged structure’ capable of growing and developing normally in a pro-inflammatory environment. While the peri-implantation-stage embryo has an attenuated immune response to inflammatory cytokines, type I interferon and type II interferon receptors are present in the primitive placenta, with some receptors appearing as early as 8 days post-fertilization (West *et al.* 2019), thus suggesting that the embryo is capable of responding to interferon signaling from the endometrium and can mount an inflammatory response.

We aimed to characterize other inflammatory stimuli that would reflect a dysregulated uterine environment. As the peri-implantation-stage placenta has the type I interferon receptor (IFNAR), we hypothesized that the type I interferon, interferon beta (IFNB), could stimulate the trophoblast innate immune response. The role of IFNB during pregnancy is still unclear. One hypothesis asserts that low, constitutive levels of IFNB secreted by commensal bacteria within the reproductive tract are necessary for the maintenance of a healthy pregnancy (Ding *et al.* 2022). However, the overproduction of IFNB due to a viral infection or autoimmune disorder can have detrimental effects on the growth and development of the placenta and fetus (Bundhun *et al.* 2017, Yockey *et al.* 2018).

We also aimed to assess the effects of lipopolysaccharides (LPSs) on TSCs. LPSs are bacterial endotoxins that can induce a strong inflammatory response in other cell types, including the mature placenta. High levels of LPSs can also have detrimental effects on placental development and fetal health (Fricke *et al.* 2018, Brown *et al.* 2019, Duval *et al.* 2019, Fan *et al.* 2019); however, there is evidence to suggest that neither murine nor human embryonic stem cells mount an immune response to LPSs (D’Angelo *et al.* 2017). We aimed to determine whether placental cells representative of a peri-implantation embryo also have attenuated responses to LPSs.

Two of the most commonly used TSC cell lines are genetically male and female. In this study, we used the CT27 (female) and CT29 (male) TSC lines to uncover the molecular mechanisms behind the sexual dimorphism of early embryonic development and the placental innate immune response during pregnancy. There are inherent differences in fetal growth and metabolism between males and females (Wells 2007, Campbell *et al.* 2022). However, we now understand that sex influences the placental innate and adaptive immune response during pregnancy (Baines & West 2023). This sexual dimorphism leaves male fetuses vulnerable to miscarriage and stillbirth (Hassold *et al.* 1983, Trudell *et al.* 2015) as well as placental pathologies, including gestational diabetes and late-onset preeclampsia (Cooperstock & Campbell 1996, Huppertz 2008, Broere-Brown *et al.* 2020). Interestingly, even with adverse pregnancy outcomes skewing male, the primary sex ratio at birth is slightly male-biased (Orzack *et al.* 2015). We used a three-dimensional (3D) spheroid model to assess the differences in immune-stimulated CT27 (female) and CT29 (male) TSCs and STBs. Our results demonstrate inherent differences in trophoblast responses to inflammatory stimuli as well as differences in gene expression and innate immune response between cell lines.

## Materials and methods

### Trophoblast stem cell culture

TSCs were first generated by Okae *et al.* (2018) and obtained through a material transfer agreement with the RIKEN Cell Engineering Division. Tissue culture-treated plates were coated with laminin-511 (iMatrix-511, ReproCell Inc., USA) and incubated at 37°C for 1 h. TSCs were plated and cultured in TSC medium containing DMEM/F12 (Thermo Fisher USA, #11320082), 0.3% bovine serum albumin (BSA) (Fisher Scientific USA, #BP9704100), 1% ITS-X (Gibco USA, #51500-056), 0.2% fetal bovine serum (FBS) (Thermo Fisher, #16141002), 0.1 mM 2-mercaptoethanol, 50 ng/mL epidermal growth factor (Millipore Sigma USA, #E9644), 1.5 μg/mL L-ascorbic acid (Millipore Sigma, USA, #A8960), 2 μM CHIR99021 (ReproCell Inc., #04-0004), 0.5 μM A83-01 (ReproCellInc., #04-0014), 1 μM SB431542 (ReproCellInc., #04-0010), 0.8 mM valproic acid (VPA) (Millipore Sigma, #P4543), and 5 μM Y27632 (ReproCellInc., #04-0012). TSCs were cultured at 37°C in 5% CO_2_. Once TSCs, reached approximately 70% confluency, TSCs were dissociated in TrypLE (Thermo Fisher, #12563011) for 12–14 min and passaged to a new iMatrix-coated plate. To differentiate toward the STB, tissue culture-treated plates were coated with 2.5 μg/mL of mouse collagen IV (Corning, USA, #354233). TSCs were plated on new wells and given 24 h to recover in TSC medium. After 24 h, TSCs were cultured in STB medium conditions: DMEM/F12, 0.3% BSA, 1% ITS-X, 0.1 mM 2-mercaptoethanol, 205 μM Y27632, 2 μM Forskolin (Millipore Sigma, #F6886), and 4% knockout serum replacement (KSR) (Thermo Fisher, #10828010). Cells were cultured in STB medium for 5 days, and the medium was replenished every other day. To grow cells as 3D spheroids, tissue culture-treated plates were rinsed with Anti-Adherence Rinsing Solution (Stem Cell Technologies, USA, #07010). TSCs were grown in 3D stem conditions for 48 h before IFNB treatment. STBs were grown in 3D STB conditions for 4 days before IFNB treatment. All cells were used between serial passages 23–28, and care was made that cells at the same passage number were used within experiments. The absence of mycoplasma was confirmed monthly using the MycoStrip mycoplasma detection kit (Invivogen, USA, #rep-mys-50).

### Fibroblast cell culture

Primary human dermal fibroblasts (HDFs) were purchased from ATCC (PCS-201-012). HDFs were cultured in fibroblast basal medium (ATCC) supplemented with a fibroblast growth kit – low serum (ATCC, #PCS-201-041) containing 7.5 mM L-glutamine, 5 ng/mL rh fibroblast growth factor, 5 μg/mL rh insulin, 1 μg/mL hydrocortisone, 50 μg/mL ascorbic acid, and 2% FBS. Cells were cultured to 70% and passaged as needed.

### LPS and IFNB treatment

TSCs, STBs, and HDFs were treated with 250 ng/mL LPSs (Millipore Sigma, #L4391) for 6 h for RT-qPCR experiments or for 15, 30, and 60 min and 24 h for western blotting. Cells were treated with 250 U/mL IFNB (R&D Systems USA, #8499) for the same time points.

### RNA isolation and RT-qPCR

All RT-qPCR experiments were designed and run in concordance with the MIQE guidelines (Bustin *et al.* 2009). RNA was isolated from cells using a Qiagen RNeasy Mini Kit (#74104) following manufacturer’s instructions. Complementary DNA (cDNA) was generated from 500 ng total RNA using the qScript cDNA synthesis kit (QuantaBio, USA, 95047). After generation of cDNA, cDNA was diluted to 50 ng per reaction and mixed with PerfeCTa SYBR Green FastMix (QuantaBio, 101414). Primer sequences for primers used in RT-qPCRs are displayed in Table 1. For RT-qPCR, reactions were incubated at 95°C for 10 min and then underwent 40 cycles of 95°C for 15 s and 60°C for 1 min using the QuantStudio 5 PCR system (Thermo Fisher). Each reaction was conducted in duplicate. The averages of the technical duplicates were used to normalize relative expression against GAPDH. Fold change was determined against an untreated control for each cell type.

**Table 1:** Primers used for RT-qPCR.

Gene	Forward	Reverse
*TLR4*	AGA​CCT​GTC​CCT​GAA​CCC​TAT	CGA​TGG​ACT​TCT​AAA​CCA​GCC​A
*IL6*	ACT​CAC​CTC​TTC​AGA​ACG​AAT​TG	CCA​TCT​TTG​GAA​GGT​TCA​GGT​TG
*TNFA*	CCT​CTC​TCT​AAT​CAG​CCC​TCT​G	GAG​GAC​CTG​GGA​GTA​GAT​GAG
*ISG15*	CGC​AGA​TCA​CCC​AGA​AGA​TCG	TTC​GTC​GCA​TTT​GTC​CAC​CA
*ISG20*	CTC​GTT​GCA​GCC​TCG​TGA​A	CGG​GTT​CTG​TAA​TCG​GTG​ATC​TC
*IFITM1*	CCA​AGG​TCC​ACC​GTG​ATT​AAC	ACC​AGT​TCA​AGA​AGA​GGG​TGT​T
*IFITM2*	ATG​AAC​CAC​ATT​GTG​CAA​ACC​T	CCC​AGC​ATA​GCC​ACT​TCC​T
*IFITM3*	TGA​GGT​CAA​GGC​AGA​AGG​AG	CCA​ACC​ATC​TTC​CTG​TCC​CT

### Western blotting

Cells were lysed in RIPA buffer (Pierce, Thermo Fisher, #89900) with Halt Phosphatase Inhibitor Cocktail (Thermo Fisher, #78442) and cOmplete, Mini, EDTA-free Protease Inhibitor (Millipore Sigma, #11836170001). After lysis, protein was sonicated in three cycles of 15 s on/30 s off. Total protein was quantified using a Qubit Broad Range Protein Assay Kit (Thermo Fisher, #A50668). After quantification, protein was diluted to 20 μg using 4X Laemmli buffer (BioRad, USA, #1610747) and 40X dithiothreitol (DTT) (Millipore Sigma, #10708984001). Protein was denatured for 10 min and then allowed to cool to room temperature for 5 min. After denaturation, protein was electrophoresed in a 4–20% mini-protean TGX stain-free protein gel (Bio-Rad, #4568096). Protein was then transferred for 5 min using the Trans-Blot Turbo transfer system onto a nitrocellulose membrane (BioRad, #1704156). After protein transfer, membranes were blocked in 3% BSA in 1X TBS-T (BioRad, #1706435) and then incubated with primary antibodies (pERK, Cell Signaling Technology, USA, #4370; STAT1, Cell Signaling Technology, #9172; TLR4, Santa Cruz Biotechnology, USA, sc293072) overnight at 4°C. After incubation, membranes were washed three times in TBS-T and then incubated for 1 h at room temperature with an infrared dye-conjugated secondary antibody. Membranes were imaged using a LICORbio Odyssey imaging system. Beta-actin (ACTB) (Cell Signaling Technology, #3700S) or GAPDH (Cell Signaling Technology, #2118S) was used to normalize protein in cell lysates. Densitometry semi-quantitative analysis was performed using ImageJ software, and relative density was calculated as a percentage of control protein after normalization.

### RNA sequencing

Purified RNA was sent to Novogene for library construction and sequencing. Briefly, messenger RNA (mRNA) was extracted from total RNA by poly-T oligo-attached magnetic beads. mRNA was subject to library preparation, and cDNA was multiplexed by PCR amplification. Libraries were quantified using Qubit, and size distribution was analyzed using the Bioanalyzer 2100 system (Agilent, USA). Quantified libraries were pooled and sequenced using the Illumina NovaSeq X Plus platform.

Raw fastq reads were trimmed to remove adapters and low-quality reads using fastp. Clean reads were mapped to the human genome (hg38), and individual mapped reads were adjusted to provide fragments per kilobase per transcript sequence per million base pairs sequenced (FPKM). Differential expression gene analysis was performed using the DESeq2 R package (1.20.0). Genes were deemed differentially expressed if they provided a false discovery rate of <0.05 and a log2foldchange >1 and <−1. Comparison of data sets (i.e. TSCs vs IFN-TSCs and STBs vs IFN-STBs) to determine differential gene expression between data sets was performed using the DESeq2 R package (1.20.0). Genes were determined to be preferentially enriched in a cell type if they had an adjusted *P*-value (padj) < 0.05 and a log2foldchange >1 or <−1. Gene ontology (GO) enrichment analysis of differentially expressed genes was performed using the clusterProfiler R package. GO terms with padj < 0.05 were considered significantly enriched.

### Statistical analysis

For molecular experiments, at least three independent biological replicates were used for each experiment, and experiments were repeated at least three times. Statistical significance was evaluated with ANOVA with Tukey’s test for multiple comparisons. All statistics were generated using GraphPad Prism 9 software. *P*-values less than 0.05 were considered statistically significant.

## Results

### LPS induces a limited inflammatory response in TSCs and STBs compared with IFNB

**Figure 1:**
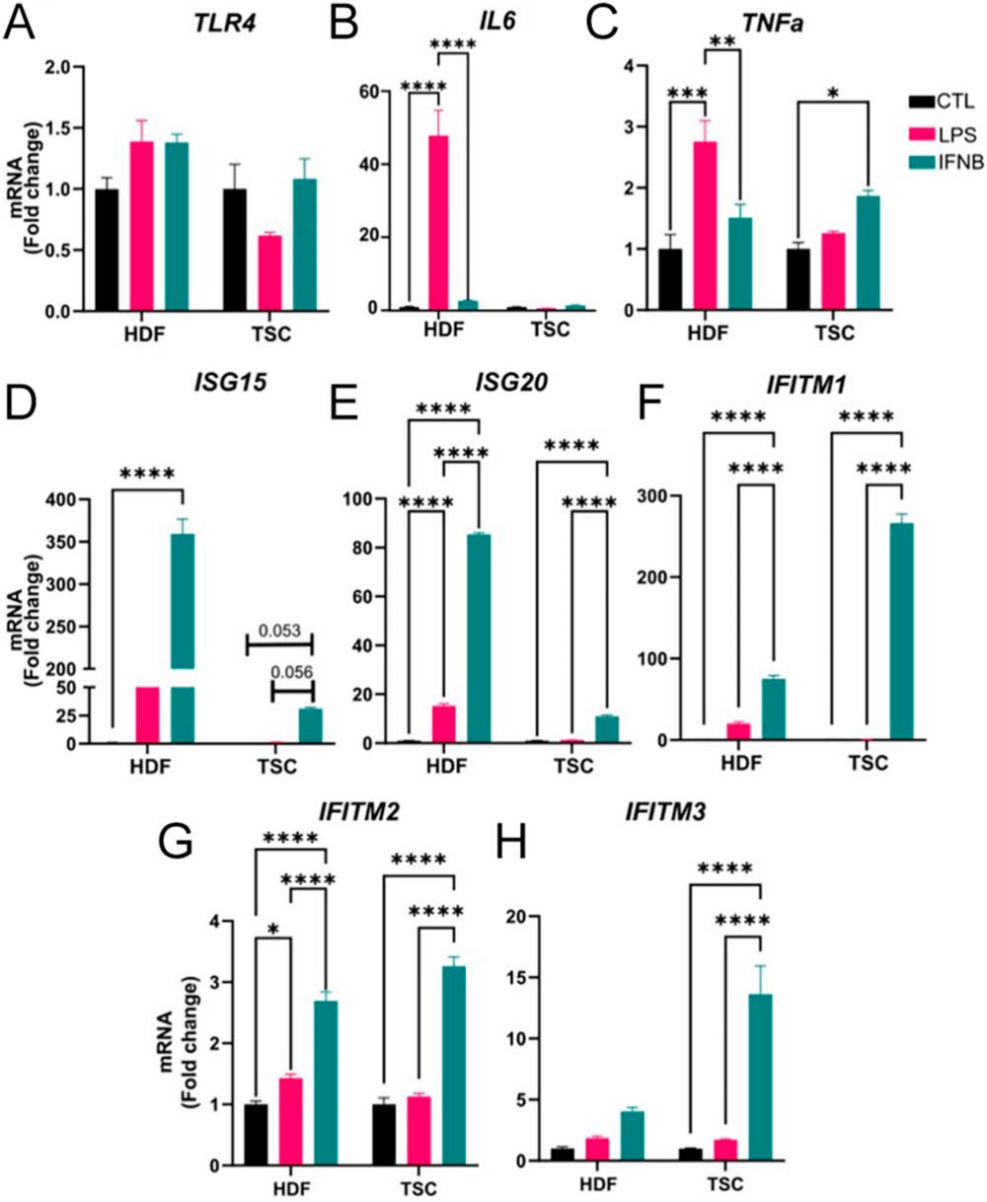
Pro-inflammatory cytokine and ISG mRNA levels in LPS- and IFNB-treated TSCs. While there is no increase in (A) pattern recognition receptor *TLR4,* LPS treatment significantly increased mRNA levels of the (B) pro-inflammatory cytokine *IL6* and (C) pro-inflammatory cytokine *TNFa* in HDFs but not TSCs. However, LPS treatment did stimulate a modest, but significant increase in *TNFa* in the TSCs. Alternatively, IFNB treatment induced an ISG response in the HDFs and TSCs for (D) *ISG15,* (E) *ISG20,* (F) *IFITM1,* and (G) *IFITM2.* Only the TSCs had increased levels of (H) *IFITM3* in response to IFNB. *P < 0.05, **P < 0.01, ***P < 0.005, ****P < 0.001.

We first measured the mRNA levels of *TLR4* and the pro-inflammatory cytokines interleukin-6 (*IL6*) and tumor necrosis factor (*TNF*). We also measured mRNA levels of the interferon-stimulated genes (ISGs), *ISG15* and *ISG20*, and the interferon-inducible transmembrane genes (IFITM), *IFITM1, IFITM2,* and *IFITM3.* While LPSs triggered a significant increase in *IL6* and *TNF* mRNA levels in the HDFs, LPSs did not stimulate an increase in *TLR4, IL6,* or *TNF* in TSCs (Fig. 1A, B, C). Alternatively, TSC treatment with IFNB induced a robust response in *ISG20, IFITM1, IFITM2,* and *IFITM3* (Fig. 1E, F, G, H) and an elevated, albeit not significant, response in *ISG15* levels (Fig. 1D) compared with wild-type and LPS-treated TSCs.

**Figure 2:**
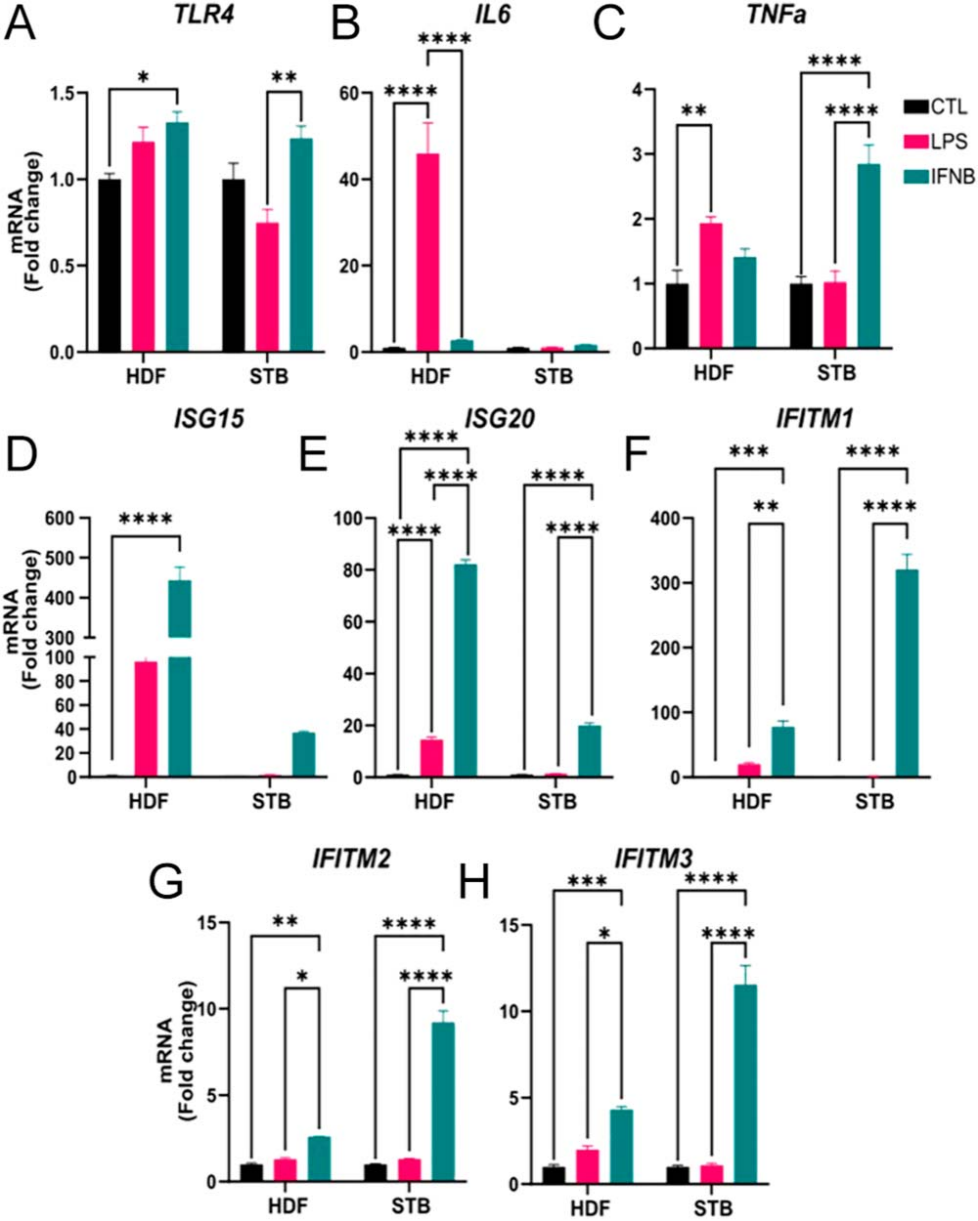
Pro-inflammatory cytokine and ISG mRNA levels in LPS- and IFNB-treated STBs. LPS treatment did not induce a significant change in the (A) pattern recognition receptor *TLR4.* However, IFNB treatment did trigger a modest but significant increase. LPS treatment significantly increased mRNA levels of the (B) pro-inflammatory cytokine *IL6* and (C) pro-inflammatory cytokine *TNFa* in HDFs but not STBs. *TNFa* levels were significantly elevated in STBs treated with IFNB. IFNB treatment also induced an ISG response in the HDFs for (D) *ISG15.* The ISGs, (E) *ISG20*, (F) *IFITM1*, (G) *IFITM2*, and (H) *IFITM3*, were significantly increased in both the HDFs and STBs. *P < 0.05, **P < 0.01, ***P < 0.005, ****P < 0.001.

LPSs also failed to stimulate a pro-inflammatory or IFN response in STBs (Fig. 2A, B, C, D, E, F, G, H). However, IFNB induced a robust response in both the STBs and HDFs. *TLR4* mRNA levels were modestly but significantly higher in IFNB-treated STBs compared with LPS treated STBs (Fig. 2A). In addition, *TNFA* was also modestly but significantly higher in IFNB-treated TSCs and STBs compared with control and LPS-treated STBs. *ISG20, IFITM1, IFITM2,* and *IFITM3* were all significantly elevated in IFNB-treated STBs. These results suggest that LPS does not stimulate a pro-inflammatory response in TSCs or STBs. However, IFNB can stimulate a robust IFN response in both cell types.

**Figure 3:**
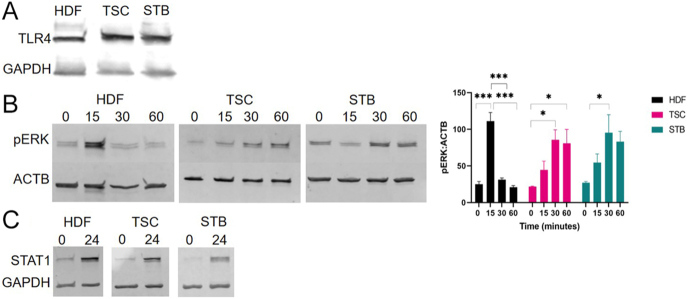
LPSs trigger rapid phosphorylation of ERK in HDFs but not TSCs or STBs. Total STAT1 is increased in all cell types after IFNB treatment for 24 h. (A) There are no differences in protein levels for TLR4 between the untreated HDFs, TSCs, and STBs. (B) Protein levels for pERK 0, 15, 30, and 60 min after LPS treatment in HDFs, TSCs, and STBs. There was a significant increase in pERK 15 min post-LPS treatment followed by a significant decrease 30 and 60 min post-treatment. Alternatively, there was a modest but significant increase in pERK 30 and 60 min post-LPS treatment in TSCs and no significant increase in pERK at any time point compared in the STBs compared with the untreated control. (C) Proteins levels for total STAT1 after 24 h of IFNB treatment. *P < 0.05, ***P < 0.005.

We next used western blotting to determine protein levels of TLR4 in the unstimulated HDFs, TSCs, and STBs and found no differences in protein abundance between the three cell types (Fig. 3A). To assess whether LPS stimulates phosphorylation of ERK1/2 in TSCs and STBs, we treated cells with LPS and then collected protein 15, 30, and 60 min after treatment. There was a delayed, modest increase in pERK in both TSCs and STBs. Conversely, there was a robust increase to LPSs at 15 min followed by a rapid decrease in pERK in the HDF control (Fig. 3B). We also used western blotting to demonstrate that IFNB treatment increased total STAT1 protein in HDFs, TSCs, and STBs after 24 h of treatment (Fig. 3C). These data further demonstrate that type I interferons trigger a robust antiviral response in TSCs and STBs, whereas LPSs do not stimulate the production of pro-inflammatory cytokines in TSCs or STBs.

### IFNB elicits a robust antiviral response in both TSCs and STBs

**Figure 4:**
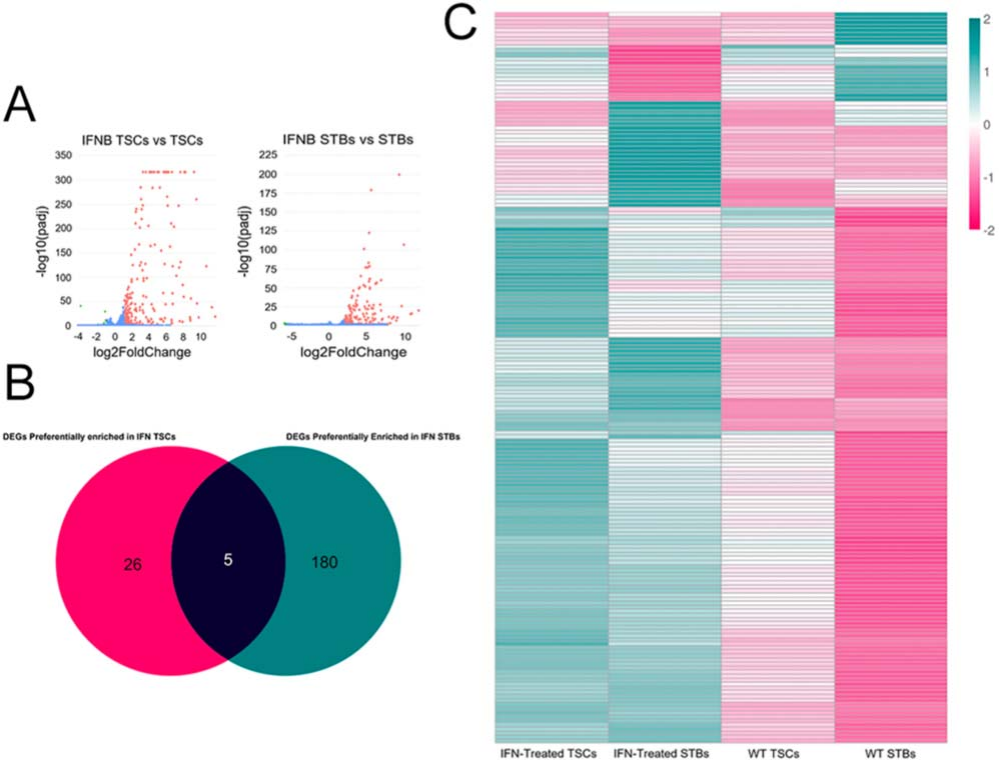
IFNB triggers an antiviral response in both TSCs and STBs, but STBs also have increased transcript levels for pro-inflammatory cytokines and chemokines and genes for recruitment of immune cells. (A) IFNB-treated TSCs had 252 (245 upregulated and 7 downregulated) DEGs compared with untreated TSCs. IFNB-treated STBs had 185 (163 upregulated and 22 downregulated) DEGs compared with untreated STBs. The red points represent transcripts with a log2foldchange >1 and an adjusted *P*-value of <0.05. The green points represent transcripts with a log2foldchange −1< and an adjusted *P*-value of <0.05. (B) Venn diagram of DEGs preferentially enriched in the IFN-treated TSCs or IFN-treated STBs after comparison between the two data sets comparing treated cells against their respective controls. (C) Heat map clustering of the preferentially enriched genes found in the IFN-treated TSCs and IFN-treated STBs. The color spectrum, ranging from teal to pink, indicates high to low normalized levels of expression of each gene.

We next compared the transcriptomes of untreated and IFN-treated TSCs and untreated and IFN-treated STBs. Using RNA-Seq, we analyzed untreated TSCs and STBs and IFN-treated TSCs and STBs. The cell types were first analyzed for gene expression, and genes with padj values < 0.05 and a log2foldchange >1 were designated as significant differentially expressed genes. There were 252 (245 upregulated and 7 downregulated) significantly different transcripts between the untreated TSCs and IFN-treated TSCs (Fig. 4A, Supplemental File 1 (see section on Supplementary materials given at the end of the article)). Similarly, there were 185 DEGs (163 upregulated and 22 downregulated) between the untreated STBs and IFN-treated STBs (Fig. 4A, Supplemental File 2). GO analysis of IFNB-treated TSCs and STBs compared with their respective controls were very similar (Supplemental Fig. 1), with GO terms related to response to virus and the type I interferon signaling pathway strongly associated with both IFNB-treated cell types. We next used GO analysis to compare differences between the TSCs and STBs and IFN-treated TSCs and IFN-treated STBs. Many pathways related to syncytialization, steroidogenesis, and protein processing were significantly enriched in the STBs and IFN-treated STBs compared with the TSCs and IFN-treated TSCs (Supplemental File 3). However, several top enriched terms for both the STBs and IFN-treated STBs compared with the TSCs and IFN-treated TSCs were related to the inflammatory response (Supplemental File 3); therefore, we next compared the DEG profiles between the TSCs and IFN-treated TSCs and STBs and IFN-treated STBs data sets to identify genes and pathways that were preferentially enriched only in the IFN-treated STBs and IFN-treated TSCs compared with the other cell types. This comparison revealed 26 genes preferentially enriched in the IFN-treated TSCs and 180 genes preferentially enriched in the IFN-treated STBs (Fig. 4B, Supplemental File 4). In addition, similar gene expression patterns were observed for five genes in both the IFN-treated TSCs and STBs (Fig. 4B, Supplemental File 4). Clustering analysis was performed to visualize differences in gene expression between each treatment group (Fig. 4C, Supplemental File 4).

GO analysis of the IFN-treated STB preferentially enriched DEGs revealed upregulation of GO terms related to response to type I interferon and response to virus (Table 2, Supplemental File 4). Many of the 180 preferentially enriched genes in the IFN-treated STBs were canonical ISGs (*STAT1, IRF1, IRF7, SOCS1, IFIT1, IFIT2, IFIT3, IFIT5, OAS1, OAS2, OAS3, ISG15, ISG20, IFI44, IFI6, IFI27, and IFI35).* In addition, genes related to viral RNA/DNA sensing and restriction machinery (*IFIH1, DDX58, TLR3, SAMHD1, ADAR, TRIM14, TRIM22, TRIM25, TRIM38, and TRIM69)* and antigen processing and the MHC class I pathway (*HLA-C, HLA-E, HLA-F, TAP1, TAP2, and NLRC5)* were upregulated in the IFN-treated STBs. Several other genes related to the inflammasome and immune cell activation (*CASP1, CASP8 TNFSF10, MYD88, and CARD11)*, and cytokines and chemokines (*CXCL16, CCL28, TNSF13B, CSF1, and PDCD1LG2)* were also elevated. This gene expression profile suggests a robust, coordinated interferon-driven antiviral and immune-activation program within the STBs. Alternatively, GO analysis revealed no upregulated GO terms within the preferentially enriched IFN-treated TSC DEGs. While there were some innate immune-related DEGs (*C3AR1, PIK3AP1, IKZF1, SIGLEC6, LY63, TRIM56, DUSP10, and FLG1)*, the gene expression profile was more representative of a heterogeneous pool of genes, suggesting a modest interferon response compared with the STBs.

**Table 2:** Top ten significant GO terms for each of BPs, MFs, and CCs identified through analysis of the preferentially enriched IFN-treated STB DEGs. A comprehensive list of all GO terms can be found in Supplemental File 4.

Category/GO term	Term ID	Adj *P*-value	Count
BP			
Response to type I interferon	GO:0034340	2.79E-45	36
Type I interferon signaling pathway	GO:0060337	5.16E-43	34
Cellular response to type I interferon	GO:0071357	5.16E-43	34
Defense response to virus	GO:0051607	5.29E-42	44
Response to virus	GO:0009615	2.70E-38	46
Defense response to other organisms	GO:0098542	3.03E-32	48
Negative regulation of viral process	GO:0048525	3.22E-28	26
Negative regulation of viral life cycle	GO:1903901	4.37E-27	24
Response to interferon-gamma	GO:0034341	1.15E-26	31
Interferon-gamma-mediated signaling pathway	GO:0060333	3.60E-26	24
CC			
MHC protein complex	GO:0042611	0.000464	5
Phagocytic vesicle membrane	GO:0030670	0.00071	7
Phagocytic vesicle	GO:0045335	0.002244	8
Recycling endosome membrane	GO:0055038	0.003444	6
Integral component of lumenal side of endoplasmic reticulum membrane	GO:0071556	0.004439	4
Lumenal side of endoplasmic reticulum membrane	GO:0098553	0.004439	4
ER to Golgi transport vesicle membrane	GO:0012507	0.00867	5
Early endosome membrane	GO:0031901	0.011595	7
Early endosome	GO:0005769	0.011595	11
Endosome membrane	GO:0010008	0.011595	13
MF			
Double-stranded RNA binding	GO:0003725	4.38E-07	10
Peptide antigen binding	GO:0042605	0.000559	5
NAD + ADP ribosyltransferase activity	GO:0003950	0.006777	4
Tumor necrosis factor receptor superfamily binding	GO:0032813	0.008414	5
Adenylyltransferase activity	GO:0070566	0.008414	4
Single-stranded RNA binding	GO:0003727	0.008414	6
Antigen binding	GO:0003823	0.008414	5
Transferase activity, transferring pentosyl groups	GO:0016763	0.008414	5
MHC protein binding	GO:0042287	0.008414	4
Tumor necrosis factor receptor binding	GO:0005164	0.009875	4

### There are inherent genetic differences between TSC lines

**Figure 5:**
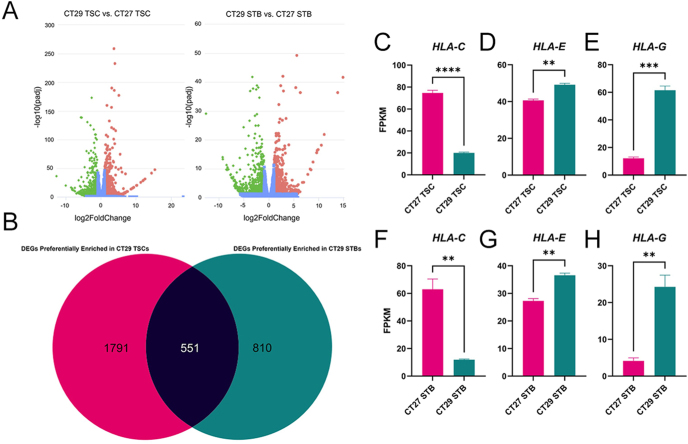
Wild-type CT27 TSCs and STBs have differences in gene expression compared with CT29 TSCs and STBs. (A) Volcano plots of DEGs. The CT29 TSCs had 1,221 upregulated and 1,121 downregulated DEGs compared with the CT27 TSCs. The CT29 STBs had 647 upregulated and 714 downregulated DEGs compared with the CT29 STBs. The red points represent transcripts with a log2foldchange >1 and an adjusted *P*-value of <0.05. The green points represent transcripts with a log2foldchange −1< and an adjusted *P*-value of <0.05. (B) Venn diagram of DEGs preferentially enriched in the CT29 TSCs or CT29 STBs after comparison between the two data sets comparing CT27 and CT29 TSCs and CT27 and CT29 STBs. (C, D, E) FPKM values of *HLA-C, HLA-E,* and *HLA-G* between the CT27 and CT29 TSCs. (F, G, H) FPKM values of *HLA-C, HLA-E,* and *HLA-G* between the CT27 and CT29 STBs. The error bars represent SEM (*n* = 3) calculated from RNA-Seq analysis. ** *P* < 0.01, ***P < 0.005, ****P < 0.001.

The experiments described in Figs 1, 2, 3, 4 were conducted using the CT29 TSC cell line; however, *Okae et al.* generated several TSC lines (Okae *et al.* 2018). As the CT29 TSCs are genetically XY, we aimed to compare the differences between XY TSCs against the XX CT27 cell line. Compared with the CT27 TSCs, the CT29 TSCs had 2,342 DEGs (1,221 upregulated and 1,121 downregulated) (Fig. 5A, Supplemental File 5). In addition, there were 1,361 DEGs (647 upregulated and 714 downregulated) in the CT29 STBs compared with the CT27s (Fig. 5A, Supplemental File 6). We next compared the gene profiles between the CT29 vs CT27 TSC and CT29 and CT27 STB differential gene expression analyses to identify genes that were preferentially enriched in each cell subtype. This comparison revealed 1,791 genes preferentially enriched in the CT29 TSCs and the 810 genes preferentially enriched in the CT29 STBs. There were 551 shared genes between the CT29 TSCs and STBs compared with the CT27s (Fig. 5B, Supplemental File 7).

We performed GO analysis on the DEGs preferentially enriched in the CT29 and CT27 TSCs and STBs. There were several significant GO terms in the CT29 TSCs (Table 3, Supplemental File 7). Many of these terms were related to cell migration and extracellular matrix organization. Alternatively, when we performed GO analysis on the DEGs preferentially enriched in the CT27 TSCs, terms related to detoxification of metals were significantly upregulated (Table 4, Supplemental File 7). Many of the upregulated genes in the CT27 TSCs belong to the metallothionein (MT) family, including *MT2A, MT1G, MT1E, MT1H, MT1F,* and *MT1X* (Supplemental Fig. 2), which are important for detoxification of metals and protection against oxidative stress (Thirumoorthy *et al.* 2007). There were no differences in gene expression of MTs between the CT27 and CT29 STBs.

**Table 3:** Top ten significant GO terms for BP and all significant CC terms identified through the analysis of the preferentially enriched CT29 TSC DEGs. A comprehensive list of the remaining significant terms can be found in Supplemental File 7.

Category/GO term	Term ID	Adj *P* value	Count
BP			
Negative regulation of cell projection organization	GO:0031345	0.010357611	17
Negative regulation of cell morphogenesis involved in differentiation	GO:0010771	0.010357611	12
Positive regulation of epithelial cell migration	GO:0010634	0.010357611	16
Negative regulation of neuron projection development	GO:0010977	0.010357611	15
Positive regulation of vasculature development	GO:1904018	0.011987787	19
Connective tissue development	GO:0061448	0.011987787	20
Cartilage development	GO:0051216	0.011987787	17
Positive regulation of endothelial cell migration	GO:0010595	0.011987787	13
Hormone catabolic process	GO:0042447	0.012879959	5
Regulation of vasculature development	GO:1901342	0.012879959	27
CC			
Extracellular matrix	GO:0031012	0.000719378	34
Proteinaceous extracellular matrix	GO:0005578	0.001110038	28
Endoplasmic reticulum lumen	GO:0005788	0.008443106	22
Anchored component of plasma membrane	GO:0046658	0.04369822	7

**Table 4:** All significant GO terms identified through the analysis of preferentially enriched CT27 TSC DEGs.

Category/GO term	Term ID	Adj *P* value	Count
BP			
Meiotic chromosome segregation	GO:0045132	0.043908046	12
Detoxification of copper ion	GO:0010273	0.043908046	5
Stress response to copper ion	GO:1990169	0.043908046	5
Cellular response to copper ion	GO:0071280	0.043908046	6
Detoxification of inorganic compound	GO:0061687	0.043908046	5
Stress response to metal ion	GO:0097501	0.043908046	5
Meiotic nuclear division	GO:0140013	0.043908046	16
Cellular zinc ion homeostasis	GO:0006882	0.047562279	7
CC			
DNA packaging complex	GO:0044815	0.002079432	14
Sodium channel complex	GO:0034706	0.008754717	6
Nucleosome	GO:0000786	0.027088395	11
Cation channel complex	GO:0034703	0.027088395	17
Voltage-gated sodium channel complex	GO:0001518	0.048745754	4

There were 11 significant pathways in the DEGs preferentially enriched in the CT29 STBs (Table 5, Supplemental File 7). Most of the terms enriched in the CT29 STBs were related to protein folding and processing. Many of the upregulated genes related to protein folding and processing belonged to the heat shock protein family (*HSPA1A, HSPA4L, HSPA1B, HSPE1, *and* HSPH1)*. Alternatively, GO analysis of the DEGs preferentially enriched in the CT27 STBs revealed several pathways related to cholesterol metabolism and steroid biosynthesis (Table 6, Supplemental File 7). Protein folding and processing, cholesterol metabolism, and steroid biosynthesis are all hallmark pathways associated with the STB; however, it appears that the CT29s and CT27s are divergent, at least at the transcriptional level, in which pathways are prioritized upon differentiation.

**Table 5:** All significant GO terms identified through the analysis of preferentially enriched CT29 STB DEGs.

Category/GO term	Term ID	Adj *P*-value	Count
BP			
Chaperone cofactor-dependent protein refolding	GO:0051085	0.022396	6
‘*De novo*’ posttranslational protein folding	GO:0051084	0.024793	6
‘*De novo*’ protein folding	GO:0006458	0.024793	7
Chaperone-mediated protein folding	GO:0061077	0.029525	8
Homeostasis of number of cells	GO:0048872	0.029525	14
Protein refolding	GO:0042026	0.039926	5
Positive regulation of protein serine/threonine kinase activity	GO:0071902	0.042084	17
Myeloid cell homeostasis	GO:0002262	0.042084	10
Positive regulation of I-kappa B kinase/NF-kappa B signaling	GO:0043123	0.042084	11
Protein maturation	GO:0051604	0.045968	16
MF			
Protein binding involved in protein folding	GO:0044183	0.028357	6

**Table 6:** Top ten significant GO terms for BP and all significant MF GO terms identified through the analysis of preferentially enriched CT27 STB DEGs. A comprehensive list of all GO terms can be found in Supplemental File 7.

Category/GO term	Term ID	Adj *P*-value	Count
BP			
Regulation of cholesterol metabolic process	GO:0090181	0.002842	8
Cholesterol biosynthetic process	GO:0006695	0.004598	8
Response to topologically incorrect protein	GO:0035966	0.004598	13
Secondary alcohol biosynthetic process	GO:1902653	0.004598	8
Response to endoplasmic reticulum stress	GO:0034976	0.004598	15
Sterol biosynthetic process	GO:0016126	0.004598	8
Response to unfolded protein	GO:0006986	0.004598	12
Regulation of response to endoplasmic reticulum stress	GO:1905897	0.005386	8
Endoplasmic reticulum unfolded protein response	GO:0030968	0.005784	10
Regulation of cholesterol biosynthetic process	GO:0045540	0.006886	6
MF			
Chaperone binding	GO:0051087	0.042609	8
Motor activity	GO:0003774	0.042609	9

Finally, both the TSCs and STBs showed differences in the major histocompatibility complex (MHC) molecules. Interestingly, there appears to be an inverse relationship between *HLA-G* and *HLA-C* expression in the CT27 and CT29 TSCs and STBs. *HLA-G* is significantly higher in the CT29 TSCs and STBs, whereas *HLA-C* is significantly higher in the CT27 TSCs and STBs. There was a modest but not significant decrease (padj = 0.07) in *HLA-E* in the CT27 TSCs and a moderate and significant decrease (*P* < 0.05) in *HLA-E* in the CT27 STBs compared with the CT29 STBs (Fig. 5C, D, E, F, G, H). Collectively, these data demonstrate that there are inherent differences in gene expression between the CT27 and CT29 TSC and STB cell lines.

### TSC cell lines respond differently to IFNB stimulation

**Figure 6:**
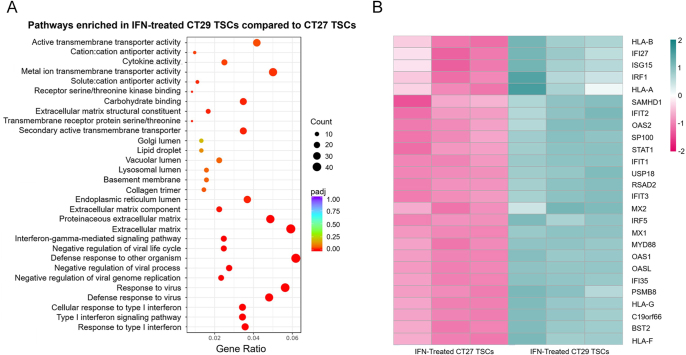
CT29 TSCs have a more robust antiviral response compared with the CT27 TSCs. (A) Gene ontology analysis comparing the IFNB-treated CT29 TSCs to the IFNB-treated CT27 TSCs revealed that pathways related to response to virus and type I interferon signaling pathway were significantly enriched in the IFNB-treated CT29 TSCs. Gene ratio refers to the ratio of significant DEGs to the total number of annotated genes within a specific GO term. (B) Heat map representation of interferon-stimulated genes and genes related to the interferon signaling pathway. The color spectrum, ranging from teal to pink, indicates high to low normalized levels of expression of each gene.

We next assessed differences between the CT27 and CT29 TSCs after treatment with IFNB. Gene ontology analysis revealed that the top GO terms enriched in the CT29 TSCs were associated with type I interferon signaling and response to virus (Fig. 6A, Supplemental File 8). We used hierarchical clustering analysis to compare differences in key genes related to the antiviral interferon response and found many of these genes were significantly increased in the IFNB-treated CT29 TSCs compared with IFNB-treated CT27 TSCs (Fig. 6B). However, these differences were restricted to the TSCs and not observed between the CT27 and CT29 STBs. Most of the pathways enriched in the IFN-treated CT29 STBs compared with the CT27 IFN-treated STBs were also enriched in the untreated CT29 STBs. These terms were mostly related to the organization of the extracellular matrix, membrane fusion, and epithelial cell proliferation, suggesting differences in differentiation potential between the CT29 and CT27 TSCs.

## Discussion

A balanced maternal immune activation response is paramount to peri-implantation placental development and embryo survival. This study demonstrates an interesting phenomenon where TSCs and STBs do not mount a pro-inflammatory cytokine response in the presence of molecules representative of a microbial infection. Messenger RNA levels of the pro-inflammatory cytokines *IL6* and *IL8* were not increased in either the TSCs or STBs after treatment with LPSs. Interestingly, when we performed western blotting to determine phosphorylation of ERK 15, 30, and 60 min after LPS treatment, we saw a delayed and sustained increase in pERK at 30 and 60 min in the TSCs and at 30 min only in the STBs. This delayed phosphorylation suggests that the classical TLR4 signaling pathway is present but potentially altered and may become responsive to LPSs in alternative circumstances. We believe that our findings support the ‘double-hit hypothesis’, which is a hypothesis that proposes that the placenta has a natural, attenuated response to microbial products but maternal viral infections can sensitize the placenta to microbial insults and induce pre-term birth (Cardenas *et al.* 2011). The studies supporting the double-hit hypothesis used human trophoblast cell lines, primary human trophoblast cells, and placentas collected from pregnant mice to demonstrate that placental cells have attenuated pro-inflammatory cytokine responses to LPSs. However, the pre-treatment of animals and cells with a viral infection sensitized cells and tissues to LPSs and led to a robust cytokine response (Cardenas *et al.* 2011). Our findings in this article support the hypothesis that the placenta has the ability to recognize LPSs through TLR4; however, the response is attenuated without an initial insult the primes that placenta to respond to bacterial products.

We did demonstrate that placental cells are responsive to type I interferons. The role of type I interferons during pregnancy and placental development is complicated and yet to be fully understood. A muted or absent type I interferon response can have detrimental effects on placental development and embryonic and fetal health. Mice lacking the type I interferon receptor (IFNAR) have diminished spiral artery remodeling, albeit the pups were only slightly growth restricted (Murphy *et al.* 2009). Loss of fetal and placental IFNAR in mice can lead to fetal viremia and increased infection-led fetal mortality compared with wild-type mice (Racicot *et al.* 2017). In addition, an active placental type I interferon response can reduce maternal viral replication in IFNAR mutant dams, suggesting that the placenta offers protection against viremia in the mother (Racicot *et al.* 2017).

While IFNAR signaling is critical for protection against viral replication in the placenta, fetus, and mother, the detrimental effects of increased IFNAR signaling appear to outweigh the benefit. Women with elevated type I interferon signaling caused by autoimmune disorders and interferonopathies are often at increased risk of maternal morbidity and mortality (Ostensen & Clowse 2013, Andrade *et al.* 2015, Meuwissen *et al.* 2016, Gupta & Gupta 2017). In the context of an active infection, Zika and other viruses induce a potent type I interferon response that disrupts placental development and induces embryonic and fetal demise (Miner *et al.* 2016, Yockey *et al.* 2018, Yockey & Iwasaki 2018). IFNAR signaling in response to Zika causes increased apoptosis in the labyrinth layer of the murine placenta and aberrations at the maternal–fetal blood barrier, leading to mixing of maternal and fetal blood within the labyrinth (Yockey *et al.* 2018). The labyrinth layer of the murine placenta is analogous to the STB layer of the human placenta. Interestingly, the treatment of chorionic villous explants with IFNB led to the formation of syncytial knots and a significant decrease in human chorionic gonadotropin subunit beta (CGB) mRNA levels (Yockey *et al.* 2018). Collectively, these previous studies suggest that most of the disruption of placental development and function occur at the STB layer.

We demonstrate that IFNB induces a robust increase in *IFITM1*, *IFITM2*, and *IFITM3* in both TSCs and STBs. This phenomenon has also been reported in immortalized choriocarcinoma cells and term villous cytotrophoblast cells (Buchrieser *et al.* 2019). In addition, high levels of the IFITM proteins inhibit STB formation and trophoblast cell invasion (Buchrieser *et al.* 2019, Zani *et al.* 2019, Degrelle *et al.* 2023), which can induce fetal demise. As the TSCs and STBs we used are molecularly representative of the peri-implantation-stage placenta, we propose that IFNB induction of IFITM proteins may activate a ‘safety switch’ mechanism to prevent the finalization of implantation and adequate trophoblast invasion in a hostile uterine environment (West *et al.* 2019). This molecular safety switch may serve to spare the potentially compromised mother from expending energy on the growth of an embryo and eventual fetus.

To comprehensively analyze transcriptional differences in IFNB stimulation between TSCs and STBs, we used a 3D spheroid model of immune stimulation in the TSCs and STBs. We opted to use a spheroid model for transcriptomics as there has been speculation that the coating substratum used to facilitate cell attachment on tissue culture-treated plates might be influencing the gene expression of TSCs (West *et al.* 2019, Logsdon *et al.* 2024). By allowing the TSCs and STBs to grow and organize in suspension culture, we removed the influence of the coating substrate. Transcriptional data comparing the IFNB-treated TSC spheroids and IFNB-treated STB spheroids revealed key differences in how the cell types mount an antiviral response. While both TSC and STB spheroids respond to IFNB, the untreated STB spheroids seem primed to produce a more robust and coordinated antiviral response, potentially due to their role as the primary maternal–fetal barrier that protects the fetus from maternal–fetal transmission of pathogens. Our results suggest that TSCs and STBs can induce distinct antiviral responses, with STBs upregulating pathways that reflect antigen sensing and communication with the uterus. As the STB is the layer of the placenta that comes into direct contact with the endometrium during invasion, these data are consistent with the idea that the placenta can provide antiviral factors to assist the maternal immune response in protection against maternal viremia (Racicot *et al.* 2017).

We also provide evidence that there are inherent differences between the commonly used CT27 and CT29 TSC lines. Of relevance to the placental immune response, there were significant differences in the expression of the human leukocyte antigen (HLA) molecules, *HLA-C, HLA-E,* and *HLA-G*, between the CT27 and CT29 TSCs and STBs. While *HLA-C* was significantly reduced in both CT27 TSCs and STBs compared with the CT29s, *HLA-E* and *HLA-G* were significantly increased. Previous studies have reported increased levels of HLA-G in EVTs and in the amniotic fluid from pregnancies carrying a male fetus (Emmer *et al.* 2003, Papuchova *et al.* 2019), suggesting that there is a sexual dimorphism to the expression of HLA molecules in extraembryonic tissues. We also observed sex-specific differences in the MT gene family. Sexually dimorphic expression of MTs has been described in other tissues, with MT expression increased in female livers and kidneys (Kim *et al.* 2009, Zhang *et al.* 2012). In addition, in a pregnant mouse model, cadmium (Cd) exposure led to significantly elevated levels of *Mt1* and *Mt2* in female placentas than in male placentas and female fetal livers had five times the accumulation of Cd compared with male fetal livers, indicating increased transfer of metals across the placenta in females (Jackson *et al.* 2022).

There were also differential responses to IFNB treatment between the CT27 and CT29 TSCs. The CT29 TSCs seemingly exhibit a stronger interferon response, with several ISGs and genes related to the IFN signaling pathway significantly higher in the IFN-treated CT29 TSCs compared with the CT27 TSCs. Our results are consistent with previous findings that male placentas have a heightened ISG response after viral exposure (Bordt *et al.* 2021); however, these differences were not observed in the STBs.

We recognize that one shortcoming of this study is the small sample size. By only comparing the CT27 and CT29 TSCs, it is difficult to determine whether the observed differences were caused by inherent genetic variation or sex. While we did observe several sex-specific phenomena that have been described in other tissues in our cell lines, further research with a larger sample size is necessary to make more substantive conclusions. Our main purpose in comparing the cell lines was to identify differences that potentially make one cell line better than another to address different hypotheses related to the placental innate immune response. Our data also reinforce the inclusion of sex as a biological variable for *in vitro* work. Future work should address the limited sample size in our study by including sex in the analyses of clinical samples or *in vivo* animal models.

Our results reinforce a growing body of work demonstrating the complexity of the peri-implantation placental immune response. Implantation failure is often attributed to an unknown etiology; however, the role of the maternal immune milieu can no longer be overlooked as a potential culprit. By responding to IFNB but not LPSs, the TSCs and STBs underscore the importance in the host response to microbial infections during pregnancy. A better understanding of how type I interferons disrupt placental development, especially during the peri-implantation period, creates new insights into how the uterine immune response influences embryo health. This molecular investigation of the peri-implantation period using representative stem cell models implicates the primitive placenta as a key mediator of maternal immune balance and opens new avenues to unlock the black box of early pregnancy loss.

## Supplementary materials





















## Declaration of interest

RCW is an Associate Editor for *Reproduction & Fertility* and was not involved in the review or editorial process for this paper. The other authors have no conflicts of interest to declare.

## Funding

This research was supported by the Ky Cha Award in Stem Cell Technology awarded by the American Society for Reproductive Medicine and the Auburn University Animal Health and Disease Research Fund.

## Author contribution statement

CRC performed experiments, contributed to study design and data analysis, and critically reviewed the manuscript. JB, MB, PD, and CP conducted the experiments. RCW conceived the study, performed data analysis, secured research funding for the study, and wrote the manuscript.

## References

[bib1] Andrade D, Kim M, Blanco LP, et al. 2015 Interferon-alpha and angiogenic dysregulation in pregnant lupus patients who develop preeclampsia. Arthritis Rheumatol 67 977–987. (10.1002/art.39029) 25603823 PMC4380868

[bib2] Baines KJ & West RC 2023 Sex differences in innate and adaptive immunity impact fetal, placental, and maternal healthdagger. Biol Reprod 109 256–270. (10.1093/biolre/ioad072) 37418168

[bib3] Bordt EA, Shook LL, Atyeo C, et al. 2021 Maternal SARS-CoV-2 infection elicits sexually dimorphic placental immune responses. Sci Transl Med 13 eabi7428. (10.1126/scitranslmed.abi7428) 34664987 PMC8784281

[bib4] Broere-Brown ZA, Adank MC, Benschop L, et al. 2020 Fetal sex and maternal pregnancy outcomes: a systematic review and meta-analysis. Biol Sex Differ 11 26. (10.1186/s13293-020-00299-3) 32393396 PMC7216628

[bib5] Brown AG, Maubert ME, Anton L, et al. 2019 The tracking of lipopolysaccharide through the feto-maternal compartment and the involvement of maternal TLR4 in inflammation-induced fetal brain injury. Am J Reprod Immunol 82 e13189. (10.1111/aji.13189) 31495009 PMC6899932

[bib6] Buchrieser J, Degrelle SA, Couderc T, et al. 2019 IFITM proteins inhibit placental syncytiotrophoblast formation and promote fetal demise. Science 365 176–180. (10.1126/science.aaw7733) 31296770

[bib7] Bundhun PK, Soogund MZ & Huang F 2017 Impact of systemic lupus erythematosus on maternal and fetal outcomes following pregnancy: a meta-analysis of studies published between years 2001–2016. J Autoimmun 79 17–27. (10.1016/j.jaut.2017.02.009) 28256367

[bib8] Bustin SA, Benes V, Garson JA, et al. 2009 The MIQE guidelines: minimum information for publication of quantitative real-time PCR experiments. Clin Chem 55 611–622. (10.1373/clinchem.2008.112797) 19246619

[bib9] Campbell GJ, Lucic Fisher SG, Brandon AE, et al. 2022 Sex-specific effects of maternal dietary carbohydrate quality on fetal development and offspring metabolic phenotype in mice. Front Nutr 9 917880. (10.3389/fnut.2022.917880) 35942169 PMC9356227

[bib10] Cardenas I, Mor G, Aldo P, et al. 2011 Placental viral infection sensitizes to endotoxin-induced pre-term labor: a double hit hypothesis. Am J Reprod Immunol 65 110–117. (10.1111/j.1600-0897.2010.00908.x) 20712808 PMC3025809

[bib11] Cooperstock M & Campbell J 1996 Excess males in preterm birth: interactions with gestational age, race, and multiple birth. Obstet Gynecol 88 189–193. (10.1016/0029-7844(96)00106-8) 8692499

[bib12] D’Angelo W, Gurung C, Acharya D, et al. 2017 The molecular basis for the lack of inflammatory responses in mouse embryonic stem cells and their differentiated cells. J Immunol 198 2147–2155. (10.4049/jimmunol.1601068) 28130495 PMC5321812

[bib13] Degrelle SA, Buchrieser J, Dupressoir A, et al. 2023 IFITM1 inhibits trophoblast invasion and is induced in placentas associated with IFN-mediated pregnancy diseases. iScience 26 107147. (10.1016/j.isci.2023.107147) 37434700 PMC10331461

[bib14] Ding J, Maxwell A, Adzibolosu N, et al. 2022 Mechanisms of immune regulation by the placenta: role of type I interferon and interferon-stimulated genes signaling during pregnancy. Immunol Rev 308 9–24. (10.1111/imr.13077) 35306673 PMC9189063

[bib15] Duval C, Brien ME, Gaudreault V, et al. 2019 Differential effect of LPS and IL-1beta in term placental explants. Placenta 75 9–15. (10.1016/j.placenta.2018.11.006) 30712669

[bib16] Emmer PM, Steegers EA, van Lierop MJ, et al. 2003 Levels of soluble HLA-G in amniotic fluid are related to the sex of the offspring. Eur J Immunogenet 30 163–164. (10.1046/j.1365-2370.2003.00373.x) 12648287

[bib17] Fan M, Li X, Gao X, et al. 2019 LPS induces Preeclampsia-like phenotype in rats and HTR8/SVneo cells dysfunction through TLR4/p38 MAPK pathway. Front Physiol 10 1030. (10.3389/fphys.2019.01030) 31507429 PMC6718930

[bib18] Fendereski M, Ming H, Jiang Z, et al. 2024 Mouse trophoblast cells have attenuated responses to TNF-alpha and IFN-gamma and can avoid synergic cytotoxicity of the two cytokines. J Immunol 212 346–354. (10.4049/jimmunol.2300210) 38054905 PMC10843640

[bib19] Fricke EM, Elgin TG, Gong H, et al. 2018 Lipopolysaccharide-induced maternal inflammation induces direct placental injury without alteration in placental blood flow and induces a secondary fetal intestinal injury that persists into adulthood. Am J Reprod Immunol 79 e12816. (10.1111/aji.12816) 29369434 PMC5908742

[bib20] Gupta S & Gupta N 2017 Sjogren syndrome and pregnancy: a literature review. Perm J 21 016–047. (10.7812/TPP/16-047) PMC5267941 28080954

[bib21] Hassold T, Quillen SD & Yamane JA 1983 Sex ratio in spontaneous abortions. Ann Hum Genet 47 39–47. (10.1111/j.1469-1809.1983.tb00968.x) 6838169

[bib22] Huppertz B 2008 Placental origins of preeclampsia: challenging the current hypothesis. Hypertension 51 970–975. (10.1161/HYPERTENSIONAHA.107.107607) 18259009

[bib23] Jackson TW, Baars O & Belcher SM 2022 Gestational Cd exposure in the CD-1 mouse sex-specifically disrupts essential metal ion homeostasis. Toxicol Sci 187 254–266. (10.1093/toxsci/kfac027) 35212737 PMC9154225

[bib24] Kim WY, Kim J, Park JD, et al. 2009 Histological study of gender differences in accumulation of silver nanoparticles in kidneys of Fischer 344 rats. J Toxicol Environ Health A 72 1279–1284. (10.1080/15287390903212287) 20077197

[bib25] Logsdon DM, Ming H, Ezashi T, et al. 2024 Transcriptome comparisons of trophoblasts from regenerative cell models with peri-implantation human embryosdagger. Biol Reprod 111 1000–1016. (10.1093/biolre/ioae120) 39109839

[bib26] Meuwissen ME, Schot R, Buta S, et al. 2016 Human USP18 deficiency underlies type 1 interferonopathy leading to severe pseudo-TORCH syndrome. J Exp Med 213 1163–1174. (10.1084/jem.20151529) 27325888 PMC4925017

[bib27] Miner JJ, Cao B, Govero J, et al. 2016 Zika virus infection during pregnancy in mice causes placental damage and fetal demise. Cell 165 1081–1091. (10.1016/j.cell.2016.05.008) 27180225 PMC4874881

[bib28] Mor G, Aldo P & Alvero AB 2017 The unique immunological and microbial aspects of pregnancy. Nat Rev Immunol 17 469–482. (10.1038/nri.2017.64) 28627518

[bib29] Murphy SP, Tayade C, Ashkar AA, et al. 2009 Interferon gamma in successful pregnancies. Biol Reprod 80 848–859. (10.1095/biolreprod.108.073353) 19164174 PMC2849832

[bib30] Ochoa-Bernal MA & Fazleabas AT 2020 Physiologic events of embryo implantation and decidualization in human and non-human primates. Int J Mol Sci 21 1973. (10.3390/ijms21061973) 32183093 PMC7139778

[bib31] Okae H, Toh H, Sato T, et al. 2018 Derivation of human trophoblast stem cells. Cell Stem Cell 22 50–63.e6. (10.1016/j.stem.2017.11.004) 29249463

[bib32] Orzack SH, Stubblefield JW, Akmaev VR, et al. 2015 The human sex ratio from conception to birth. Proc Natl Acad Sci U S A 112 E2102–E2111. (10.1073/pnas.1416546112) 25825766 PMC4413259

[bib33] Ostensen M & Clowse M 2013 Pathogenesis of pregnancy complications in systemic lupus erythematosus. Curr Opin Rheumatol 25 591–596. (10.1097/BOR.0b013e328363ebf7) 23917157

[bib34] Papuchova H, Meissner TB, Li Q, et al. 2019 The dual role of HLA-C in tolerance and immunity at the maternal-fetal interface. Front Immunol 10 2730. (10.3389/fimmu.2019.02730) 31921098 PMC6913657

[bib35] Racicot K, Aldo P, El-Guindy A, et al. 2017 Cutting edge: fetal/placental type I IFN can affect maternal survival and fetal viral load during viral infection. J Immunol 198 3029–3032. (10.4049/jimmunol.1601824) 28264970 PMC5633930

[bib36] Ruane PT, Garner T, Parsons L, et al. 2022 Trophectoderm differentiation to invasive syncytiotrophoblast is promoted by endometrial epithelial cells during human embryo implantation. Hum Reprod 37 777–792. (10.1093/humrep/deac008) 35079788 PMC9398450

[bib37] Sehring J, Beltsos A & Jeelani R 2022 Human implantation: the complex interplay between endometrial receptivity, inflammation, and the microbiome. Placenta 117 179–186. (10.1016/j.placenta.2021.12.015) 34929458

[bib38] Thirumoorthy N, Manisenthil Kumar KT, Shyam Sundar A, et al. 2007 Metallothionein: an overview. World J Gastroenterol 13 993–996. (10.3748/wjg.v13.i7.993) 17373731 PMC4146885

[bib39] Trudell AS, Cahill AG, Tuuli MG, et al. 2015 Stillbirth and the small fetus: use of a sex-specific versus a non-sex-specific growth standard. J Perinatol 35 566–569. (10.1038/jp.2015.17) 25789818 PMC4520769

[bib40] Turco MY & Moffett A 2019 Development of the human placenta. Development 146 dev163428. (10.1242/dev.163428) 31776138

[bib41] Wells JC 2007 Sexual dimorphism of body composition. Best Pract Res Clin Endocrinol Metab 21 415–430. (10.1016/j.beem.2007.04.007) 17875489

[bib42] West RC, Ming H, Logsdon DM, et al. 2019 Dynamics of trophoblast differentiation in peri-implantation-stage human embryos. Proc Natl Acad Sci U S A 116 22635–22644. (10.1073/pnas.1911362116) 31636193 PMC6842583

[bib43] Yockey LJ & Iwasaki A 2018 Interferons and proinflammatory cytokines in pregnancy and fetal development. Immunity 49 397–412. (10.1016/j.immuni.2018.07.017) 30231982 PMC6152841

[bib44] Yockey LJ, Jurado KA, Arora N, et al. 2018 Type I interferons instigate fetal demise after Zika virus infection. Sci Immunol 3 eaao1680. (10.1126/sciimmunol.aao1680) 29305462 PMC6049088

[bib45] Zani A, Zhang L, McMichael TM, et al. 2019 Interferon-induced transmembrane proteins inhibit cell fusion mediated by trophoblast syncytins. J Biol Chem 294 19844–19851. (10.1074/jbc.AC119.010611) 31735710 PMC6937555

[bib46] Zhang D, Jin T, Xu YQ, et al. 2012 Diurnal-and sex-related difference of metallothionein expression in mice. J Circadian Rhythms 10 5. (10.1186/1740-3391-10-5) 22827964 PMC3585924

