# Supplementary figures and images for: Trophoblast stem cells and syncytiotrophoblasts lack inflammatory responses to LPS but retain robust interferon-mediated antiviral immunity
Source: Reprod Fertil. 2026 Jul 7;7(3):RAF250176. doi: 10.1530/RAF-25-0176 (PMC13386154; doi:10.1530/RAF-25-0176)

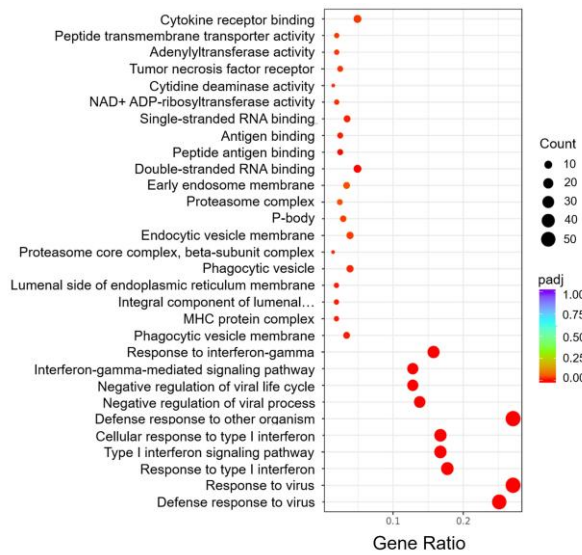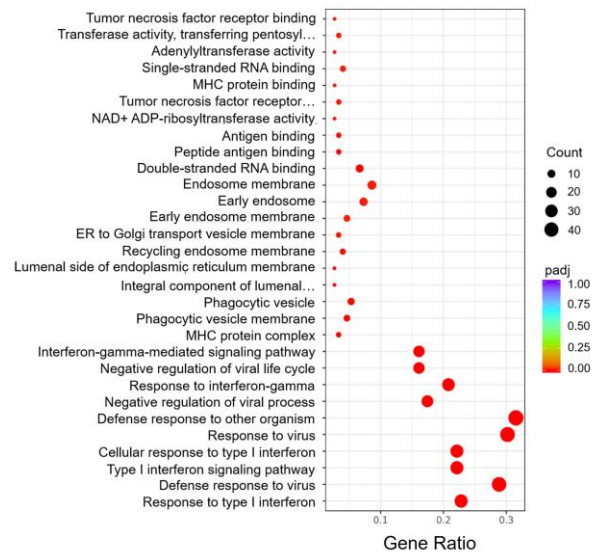

Supplement: Supplementary file 1 [file RAF-25-0176_supplementary_figure_1.pdf]

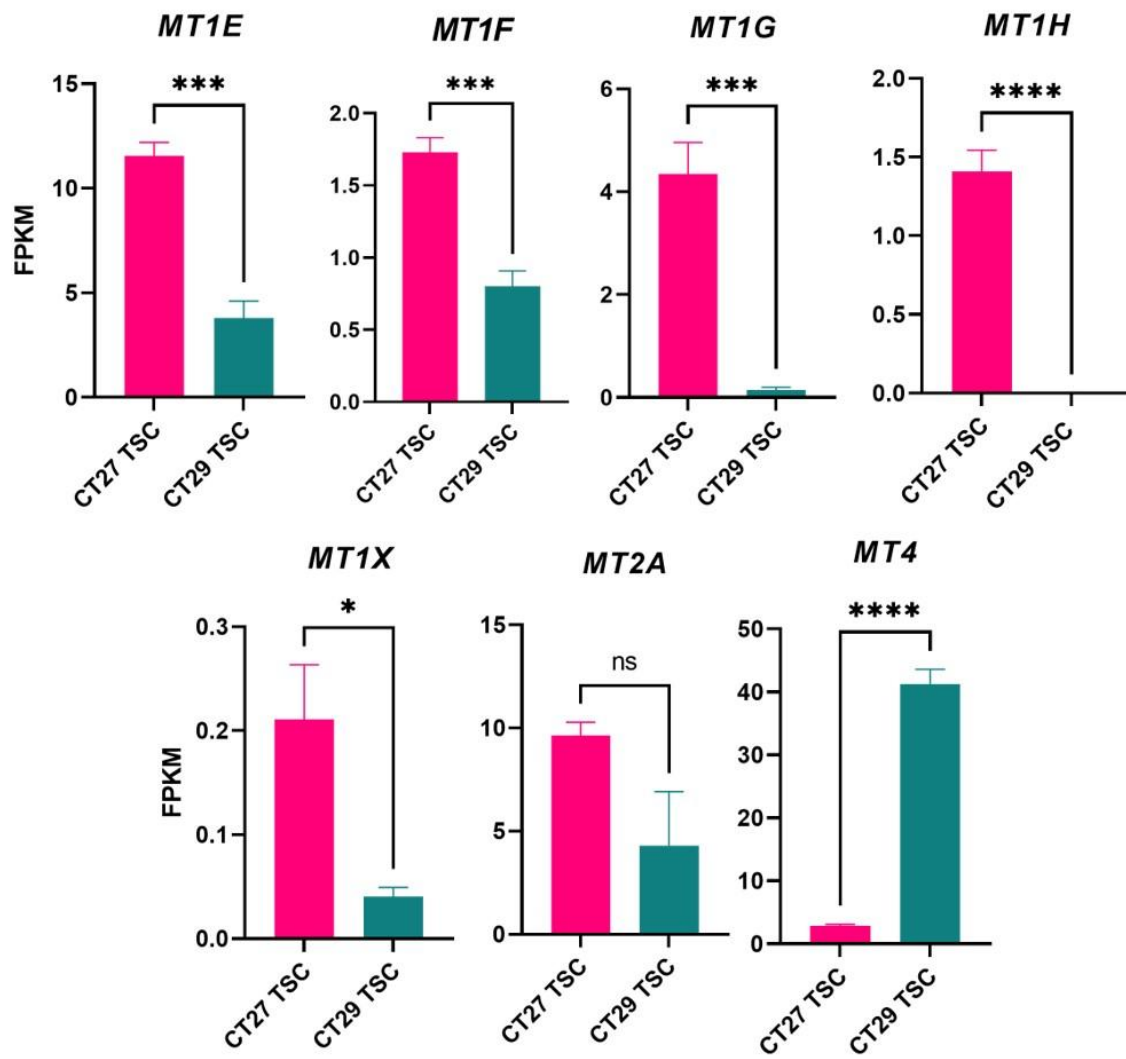

Supplement: Supplementary file 2 [file RAF-25-0176_supplementary_figure_2.pdf]
